# Study of a First Approach to the Controlled Fermentation for Lambic Beer Production

**DOI:** 10.3390/microorganisms11071681

**Published:** 2023-06-28

**Authors:** Vanesa Postigo, Margarita García, Teresa Arroyo

**Affiliations:** 1 Department of Agri-Food, Madrid Institute for Rural, Agriculture and Food Research and Development (IMIDRA), El Encín, A-2, Km 38.2, 28805 Alcalá de Henares, Spain; margarita_garcia_garcia@madrid.org (M.G.); teresa.arroyo@madrid.org (T.A.); 2 Brewery La Cibeles, Petróleo 34, 28918 Leganés, Spain

**Keywords:** non-*Saccharomyces*, lambic, *Brettanomyces*, flavour

## Abstract

Non-*Saccharomyces* yeasts represent a great source of biodiversity for the production of new beer styles, since they can be used in different industrial areas, as pure culture starters, in co-fermentation with *Saccharomyces*, and in spontaneous fermentation (lambic and gueuze production, with the main contribution of *Brettanomyces* yeast). The fermentation process of lambic beer is characterized by different phases with a characteristic predominance of different microorganisms in each of them. As it is a spontaneous process, fermentation usually lasts from 10 months to 3 years. In this work, an attempt was made to perform a fermentation similar to the one that occurred in this process with lactic bacteria, *Saccharomyces* yeast and *Brettanomyces* yeast, but controlling their inoculation and therefore decreasing the time necessary for their action. For this purpose, after the first screening in 100 mL where eight *Brettanomyces* yeast strains from D.O. “Ribeira Sacra” (Galicia) were tested, one *Brettanomyces bruxellensis* strain was finally selected (B6) for fermentation in 1 L together with commercial strains of *Saccharomyces cerevisiae* S-04 yeast and *Lactobacillus brevis* lactic acid bacteria in different sequences. The combinations that showed the best fermentative capacity were tested in 14 L. Volatile compounds, lactic acid, acetic acid, colour, bitterness, residual sugars, ethanol, melatonin and antioxidant capacity were analysed at different maturation times of 1, 2, 6 and 12 months. Beers inoculated with *Brettanomyces* yeast independently of the other microorganisms showed pronounced aromas characteristic of the *Brettanomyces* yeast. Maturation after 12 months showed balanced beers with “Brett” aromas, as well as an increase in the antioxidant capacity of the beers.

## 1. Introduction

Consumer demand for new flavours has not only focused research on finding new ingredients, but also on using these ingredients to brew different styles of traditional beers from other countries. Of particular interest are the sour and lambic styles, which are not popular styles in Spain, however, we are increasingly finding consumers who want to try new styles.

Sour beers can be encompassed in several styles that have been produced and marketed since ancient times, which is the reason that many breweries have focused their production of new beers on the revival of these styles [1,2]. Sour beers can be produced under different approaches. Traditionally, these beers are fermented spontaneously by allowing different acidifying microorganisms to act, such as yeasts of the genus *Brettanomyces*, acetic and/or lactic acid bacteria (LAB) [3]. The interactions between the different microorganisms will determine the complex flavour profiles of these beers. Sour beers have a sour sensory taste mainly due to the production of lactic and acetic acid by the microorganisms involved in brewing [4]. Furthermore, these beers show a fruity character due to esters such as ethyl acetate, ethyl hexanoate and isoamyl acetate [5]. The most notable examples of sour beers are Belgian lambics and Flanders red ales, German Berliner Weisse and Sour ales. Lambic beers are traditionally brewed in or near the Sene River valley, an area near Brussels (Belgium). These regions are considered to have the necessary microbiology for such fermentations. Brewing for the production of lambic traditionally takes place only during the colder months of the year (October to March), since cold nights are needed to lower the wort temperature to about 20 °C in one night. The mixed fermentation and maturation process can proceed for up to three years and is characterized by a microbial succession of different yeast and bacterial species [6]. Previous studies of the lambic beer fermentation process identified four phases [7]: the *Enterobacteriaceae* phase (starts after three-seven days and proceeds until 30–40 days) [8], the main fermentation phase characterised by the fermentation of yeast strains such as *Saccharomyces cerevisiae* and *Saccharomyces pastorianus* (starts after three-four weeks of fermentation) [3,9], the acidification phase characterised by the presence of *Pediococcus* spp. and occasionally *Lactobacillus* spp. (after three-four months of fermentation) [10,11], in the middle *Brettanomyces* spp. became also prevalent (after four-eight months) and finally the maturation phase (after 10 months) where the wort gradually attenuated and is characterised by a decrease in LAB. Enterobacteria generally grow during the early stages of fermentation traditional lambic beer and can produce undesirable aromas as well as certain long-chain fatty acids that contribute to the initial formation of the lambic beer aromatic profile [12]. *Saccharomyces* species is responsible for degrading most of the carbohydrates present in the wort into ethanol and carbon dioxide, thus being responsible for the major decrease in attenuation during the whole lambic brewing process [9,13]. Although LAB are one of the spoilage microorganisms that are generally undesirable in beer and resistant to the antimicrobial properties of hops, they are, together with acetic acid bacteria, responsible for the acidification of lambic beers. They are therefore indispensable for providing the characteristic acidity of this type of beer [3,7,14,15,16]. *Brettanomyces* species will be responsible for producing higher attenuation, and even over-attenuation, as well as producing the characteristic flavour (unique phenolic and ester profiles) of lambic beer. Due to their nutritionally efficient metabolism, *Brettanomyces* species are able to survive the entire lambic beer production process, unlike *Saccharomyces* species which cannot [17,18,19].

It has also been shown that the different microorganisms involved in beer brewing can produce certain compounds, as well as provide antioxidant capacity to the beer [20,21]. One of these compounds is melatonin, which is a hormone able to regulate circadian cycles in mammals [22]. Therefore, under moderate consumption, they can be considered functional foods as these compounds have antioxidant [23,24], anti-ageing [25,26], anti-inflammatory [27,28], anti-tumour [29,30] and metabolic properties [31,32].

Breweries, apart from the traditional Belgian breweries [4,9], do not generally brew beers with spontaneous fermentation, due to the difficulties of control and the time required to obtain them [33]. As it is a spontaneous fermentation, the proliferation of the different microorganisms is successive throughout the fermentation process, thus prolonging it over time. This is the reason that the aim of this study has focused on a first approach to lambic brewing by means of the different combinations of microorganisms (lactic acid bacteria, *Saccharomyces* yeast and *Brettanomyces* yeast) that may be involved in lambic brewing and thus control and reduce the production time of these beers.

## 2. Materials and Methods

### 2.1. Yeast Strains and Bacteria

Eight different *Brettanomyces* strains were used in this study (Table 1), two of them commercial, while the remaining six were isolated from different viticultural environments belonging to D.O. “Ribeira Sacra” (Galicia, Spain). They were stored under cryopreservation at −80 °C (YPD broth supplemented with 40% (*w*/*v*) glycerol). The *Saccharomyces* yeast used in the trials corresponds to the commercial strain S-04. Likewise, the strain of lactic acid bacteria used was the commercial strain of the species *Lactobacillus brevis*.

### 2.2. Brettanomyces Yeast Preselection

The wort used to perform the different trials was brewed at the La Cibeles brewery (Madrid) and had the following characteristics: pH 5.2; gravity, 11.10 °Plato and 1.047 g cm^−3^; free amino nitrogen, 232.15 ppm and bitterness, 33.55 IBU.

The eight *Brettanomyces* yeast strains were tested in a pure culture fermentation at lab scale (100 mL) in order to test their fermentative capacity. The inoculum was grown using YPD broth medium (1% bacteriological peptone, 2% yeast extract and 2% glucose; all *w*/*v*) (Condalab, Madrid, Spain) at 28 °C for 24 h and inoculation levels for the strains were 10^6^ cells mL^−1^. Fermentation was performed in triplicate at 20 °C, with 100 rpm shaking and anaerobiosis, in a vertical incubator equipped with shaking plates. Fermentation was monitored by daily weight loss.

### 2.3. Experiments Performed in 1 L

In the traditional method of processing, there is a natural succession of the different microorganisms, producing competition between them. However, in modern fermentation, it is possible to control the different phases of microbial activity, which makes it possible to introduce first those microorganisms that are less competitive. This allows them to act on the simplest sugars and thus establish their population in the absence of better competing microbes.

**Figure 1:**
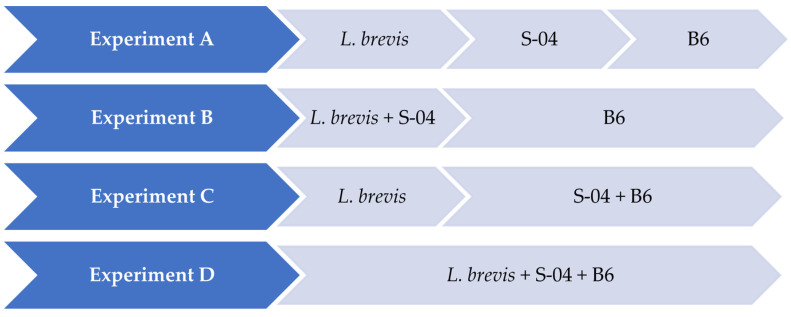
Fermentations performed in 1 L under different test combinations.

In a second phase, after having selected the most suitable strain to conduct the fermentations, it was tested in 1 L fermentations under different test combinations (Figure 1).

- Experiment A (sequential fermentation): *Lactobacillus brevis* + S-04 + B6. Fermentation was performed sequentially, with the bacteria *L. brevis* being inoculated first, after three days the yeast strain *Saccharomyces* S-04 was inoculated and finally after four days, when fermentation activity was starting to decrease, the *Brettanomyces bruxellensis* B6 strain was added.
- Experiment B: (*Lactobacillus brevis* + S-04) + B6. First, the *Saccharomyces* yeast was inoculated together with the lactic acid bacteria. After three days the *B. bruxellensis* ((RS17/CR028) strain was inoculated.
- Experiment C: *Lactobacillus brevis* + (S-04 + B6). First, the *L. brevis* bacteria was added. After three days of fermentation, the S-04 strain and *B. bruxellensis* strain were inoculated.
- Experiment D (mixed fermentation): *Lactobacillus brevis* + S-04 + B6. The microorganisms were inoculated at the same time.

Fermentation conditions were 20 °C, aerobically, no stirring and the following concentration of microorganism, *S. cerevisiae* (S-04) and *B. bruxellensis* (B6) 10^6^ cells mL^−1^ and for *L. brevis* 10^8^ cells mL^−1^ [34]. For *Saccharomyces* and *Brettanomyces* yeast strains, the inoculum was prepared as for the fermentations in 100 mL (Section 2.2), while for *L. brevis* the inoculum was incubated under static conditions for 72 h in MRS broth medium (Condalab, Madrid, Spain) at 37 °C. Fermentation was also monitored daily by weight loss in a thermostable room (14 °C). Samples were taken daily to analyse the production of lactic acid and acetic acid. After stabilisation of the different fermentations (similar weight after two consecutive days), bottling was performed in 33 cL brown glass bottles with 7 g L^−1^ glucose (sterilized under UV light). The maturation of the beers was performed at 14 °C to promote the action of *Brettanomyces* yeast on the beer.

### 2.4. Fermentation in 14 L

Once the 1 L fermentations were completed, the combinations that produced adequate levels of ethanol, acetic acid and lactic acid in less than two weeks were selected. Then, to contrast these results and the sensory characteristics of the beer produced, the trials were reproduced on a larger scale (14 L). In this case, the fermentations were performed in 20 L cylindrical steel vats containing 14 L of wort (directly transferred after processing and cooling). The temperature was maintained at 20 °C by immersion of the fermenters in recirculation cooled water tanks. Fermentation evolution was controlled daily by density measurement until stabilisation. Samples of the fermentations were also taken daily to analyse the evolution of the yeast and bacteria populations. After appropriate dilutions according to the population evolution, the samples were plated in the Petri dishes easySpiral equipment (Interscience, Saint-Nomla-Bretèche, France), in the different specific media (YPD + Chloramphenicol, MRS + Natamycin, and *Brettanomyces* agar (Condalab, Madrid, Spain)). YPD + Chloramphenicol was used for *Saccharomyces* yeast monitoring and *Brettanomyces* agar for *Brettanomyces* yeast monitoring. Both agar plates were incubated at 28 °C for three—four days. MRS + Natamycin was used for bacteria monitoring and plates were incubated at 37 °C for one week. Bottling and maturation were performed following the same pattern as for the 1 L fermentation. In order to study the evolution of the different beer components, the beer was analysed after one month, two months, six months and one year for aroma production, melatonin production, antioxidant capacity and CDR FoodLab analysis. Finally, the beers were tasted after one year.

### 2.5. Beer Analysis

The daily analyses performed during fermentation of the beer, as well as after maturation, were performed with the equipment CDR FoodLab based on enzymatic reactions and spectrophotometry (https://www.cdrfoodlab.com/analysis-systems/cdrbeerlab/) (accessed on 10 May 2023). The general parameters that were analysed in the beers are as follows: colour (range 1–100 EBC-European Brewing Convention), bitterness (range 5–100 IBU—International Bitterness Unit), lactic acid (range 150–3500 ppm), acetic acid (range 20 and 220 ppm), ethanol (range from 0 to 10% (*v*/*v*)) and residual fermentable sugars (glucose, fructose and maltose) (range from 0.1 to 18 g L^−1^).

The samples were degassed through a cellulose filter of grade 2 V (Whatman, Maidstone, UK) prior to analysis.

### 2.6. Aromatic Compounds

Thirty-three volatile compounds belonging to the following groups of aromatic compounds were analysed: higher alcohols, esters, acids, aldehydes-ketones, lactones and phenols. Their determination was performed in accordance with the method of Ortega et al. [35] based on liquid-phase microextraction with dichloromethane (DCM) (Panreac-Applichem, Barcelona, Spain) and then identified by gas chromatography (GC).

The extraction of the different samples was performed in triplicate. Determination of the major aromatic compounds was performed by using four different internal standards (200 ppm each one) (2-butanol, 4-methyl-2-pentanol, 4-hydroxy-4-methyl-2-pentanone, 2-octanol).

Analyses were performed using a gas chromatograph 6850 (GC-FID, Agilent Technologies, Inc., Santa Clara, CA, USA) equipped with a flame ionization detector. A DB-WAX column was used (60 m × 0.32 mm i.d. and 0.5 µm film). The injector and detector temperature were 200 °C, with splitless injection. The initial column temperature was 40 °C (5 min), progressively increasing by 3 °C min^−1^ until 200 °C was reached. The carrier gas was helium at a flow rate of 2 mL min^−1^.

### 2.7. Melatonin Production

The determination of melatonin in the matured beer was performed by solid phase extraction (SPE) using RP-18 standard PP-tubes (Agilent Technologies, Inc., Santa Clara, CA, USA).

Prior to the extraction, the columns were conditioned with 2 mL of methanol (Scharlab, Barcelona, Spain) and 5 mL of Milli-Q water. Subsequently, they were loaded with 500 μL of beer, the impurities were washed with 2 mL of Milli-Q water and eluted with 2 mL of methanol [36]. The samples were then dried using a stream of nitrogen and a thermoblock at 80 °C. Finally, the extract was reconstituted in 300μL of methanol (Panreac-Applichem, Barcelona, Spain) and 700 μL of mobile phase (formic acid (0.1%)/Acetonitrile (95:5)) (HPLC grade; Carlo Erba, Italy/Panreac-Applichem, Barcelona, Spain). The reconstituted extracts were filtered (0.22 μm) and transferred to 1.5 mL amber tubes before HPLC analysis.

The equipment used for the chromatographic determination was a Waters 600 HPLC controller system together with a Waters 717 plus autosampler and equipped with a Waters 2475 multifluorescence detector. The wavelengths set on the fluorescence detector were 270 nm for excitation and 372 nm for emission. The analyses were performed with a ZORBAX Eclipse Plus C18 column (Agilent Technologies, Inc., Santa Clara, CA, USA). The solvent was a mixture of 0.1% formic acid in water and acetonitrile (95:5) at a flow rate of 1 mL min^−1^. The column and detector temperature were 30 °C. The melatonin concentration was determined by a linear calibration curve (R^2^ > 0.9856) [37,38,39].

### 2.8. Antioxidant Capacity

After the different maturation times, the e-BQC lab device was used to determine the antioxidant capacity of the beers (Bio-quochem, Asturias, Spain, www.bioquochem.com) (accessed on 10 May 2023). The measurements made by the equipment are based on the redox potential of the sample and are expressed in micro-Coulombs (µC) [40]. The e-BQC lab can distinguish between fast acting antioxidants (Q1) (uric acid, ascorbic acid (vitamin C), GSH, vitamin E, CoQ10, carotenoids…) and slow acting antioxidants (Q2) (polyphenols, alpha-lipoic acid (ALA), resveratrol, astaxanthin…).

The antioxidant capacity was determined by calculating the concentration by the TEAC (Trolox Equivalent Antioxidant Capacity) assay using a solution of 6-hydroxy-2,5,7,8-tetramethylchroman-2-carboxylic acid (Trolox 8 mM L^−1^) (Sigma Aldrich, Vienna, Austria) in methanol 5% (Panreac-Applichem, Barcelona, Spain) and pH 4.5.

A calibration curve for Q1 (R^2^ = 0.9974) and Q2 (R^2^ = 0.9876) was performed using successive dilutions of Trolox (50, 100, 200, 400 and 800 µM). The results of the antioxidant capacity of the beers brewed were expressed as millimoles of Trolox equivalents per litre (mmol TE L^−1^). Samples were measured at room temperature (20–22 °C).

### 2.9. Sensory Analysis

Beers brewed at both the 1 L and 14 L scale were evaluated by a panel of ten trained and experienced tasters, ranging in age from 35 to 62 years old (five women and five men). The tastings were performed in a dedicated tasting room, with individual booths and a room temperature of 24 to 25 °C [41]. The attributes that were analysed by the tasters were classified into three groups: appearance (colour, foam retention), smell (esters, alcohols) and taste (alcohol, sweet, salty, acidic, bitterness, astringency, effervescence, warmth, slickness, body). The panellists used a scale from zero (attribute not perceptible) to five (attribute strongly perceptible) to rate the intensity of each attribute. The overall impression was also evaluated by each panellist, taking into account visual, aromatic and gustatory aspects, as well as the lack of defects [42]. The main mean values of the different attributes of each of the beers were plotted on a radar chart.

### 2.10. Statistical Analysis

Means and standard deviations were calculated for the results obtained from the chemical and aromatic analyses of the beers at the different scaling and maturations. The different maturation periods of experiments A and B were subjected to a one-way analysis of variance (ANOVA) with Tukey’s post hoc test at a significance level of *p* < 0.05. A principal component analysis (PCA) was performed by combining the different experiments (A and B) and the main analyses performed (total higher alcohols, total esters, total aldehydes/ketones, total fatty acids, γ-butyrolactone, guaiacol, bitterness, residual sugars, ethanol, lactic acid, acetic acid, colour, melatonin and antioxidant capacity) to group those samples with the greatest similarities. Statistical analyses were performed with R Studio 4.1 (Integrated Development for R. RStudio, PBC, Boston, MA, USA).

## 3. Results and Discussion

### 3.1. Brettanomyces Yeast Preselection

The study of lambic style beers was performed on an initial selection of eight yeast strains of the genus *Brettanomyces*. Two of them were commercial (*Brettanomyces bruxellensis* and *Brettanomyces lambicus*) and six were isolated in Galicia (D.O.P. “Ribeira Sacra”) belonging to the species *B. bruxellensis* (RS15/CR002_1 (B1), RS16/CR001-T2_1 (B2), RS16/CR003_1 (B3), RS16/CR003-T2 (B4), RS17/CR020 (B5) and RS17/CR028 (B6)).

**Figure 2:**
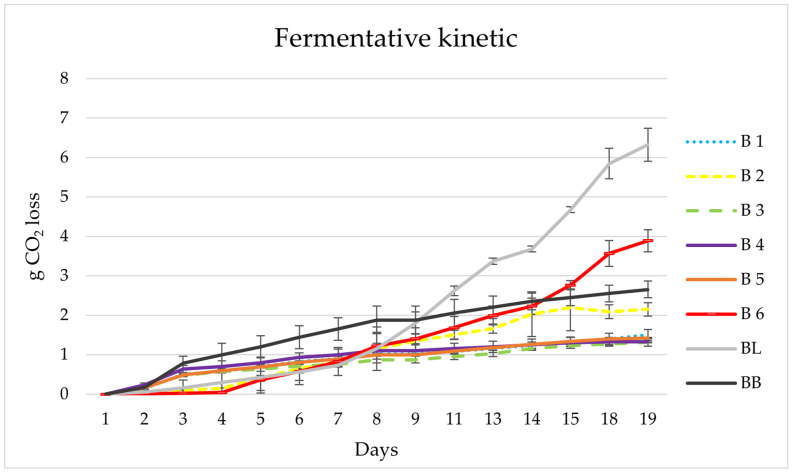
Fermentation kinetics of the *Brettanomyces* strains fermented in 100 mL. BB, commercial *B. bruxellensis*; BL, commercial *B. lambicus*; B1, RS15/CR002_1; B2, RS16/CR001-T2_1; B3, RS16/CR003_1; B4, RS16/CR003-T2; B5, RS17/CR020 and B6, RS17/CR028.

The first selection was made by determining the fermentative capacity of the yeast stains in 100 mL of wort (Figure 2). The genus *Brettanomyces* possesses the ability to ferment monosaccharides, disaccharides, trisaccharides, dextrins and even starch [43,44]. However, it has a slower fermentation rate than other species such as *Saccharomyces*. All strains showed similar fermentation kinetics reaching stability at around day 19 (from 1.33 to 6.33 g CO_2_ loss), with strains BB (2.65 g CO_2_), BL (6.33 g CO_2_), B2 (2.15 g CO_2_) and B6 (3.89 g CO_2_) showing the highest CO_2_ loss. After fermentation was finished, the resulting beers were analysed by direct olfaction to select the one with the characteristic aromas of *Brettanomyces* yeasts (animal, barnyard, sweaty horse, medicinal, smoky) [45] and no unpleasant aromas.

According to these results, strain *B. bruxellensis* B6 (RS17/CR028) was selected because despite having a fermentation capacity of about half that of the commercial strain BL (*B. lambicus*), it showed more fruit aromas than the commercial strain.

### 3.2. Lab Scale Fermentations 1 L

**Figure 3:**
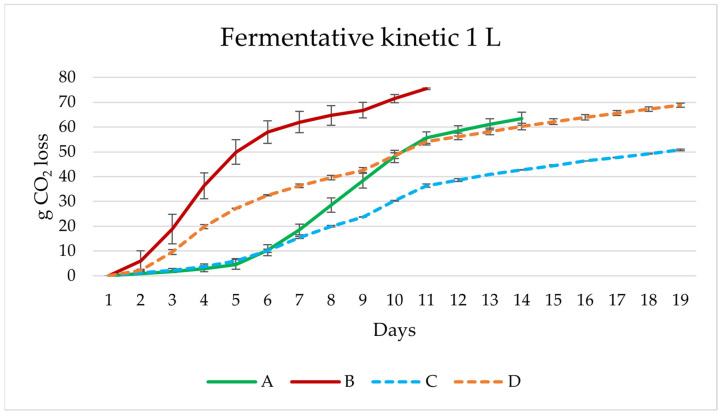
Fermentation kinetics of the different experiments with *B. bruxellensis* B6, *L. brevis* and S-04 *Saccharomyces* strain fermented in 1 L. A, sequential experiment; B, (mixed *L. brevis* + S-04) + B6; C, *L. brevis* + (mixed S-04 + B6); D, mixed *L. brevis* + S-04 + B6.

The next scale-up consisted of testing the selected 100 mL *B. bruxellensis* yeast strain (B6) under four different combinations (Figure 1), with the lactic bacteria *Lactobacillus brevis* and the yeast *Saccharomyces* S-04 strain. Growth kinetics of the different experiments in 1 L (A, B, C, D) are shown in Figure 3.

The fastest fermentations were those performed in experiment A and B, since stabilisation was at day 14 and 11, respectively, while experiments C and D took longer, with the fermentative capacity of C being lower than the rest. However, experiments A and C with lactic acid bacteria as the initial inoculum showed a slowdown in fermentation, possibly due to incomplete adaptation of the bacteria to the hopped wort [34] or the temperature of fermentation. In contrast, experiment B and D, where the lactic acid bacteria (LAB) were fermenting together with S-04 or S-04 and B6 yeast, produced a more vigorous fermentation. The interactions between LAB and yeasts are complex. The growth and metabolic rate of LAB may decrease, increase or remain unaffected in the presence of yeast, depending on several factors such as the matrix or the strains of microorganisms used [46].

**Figure 4:**
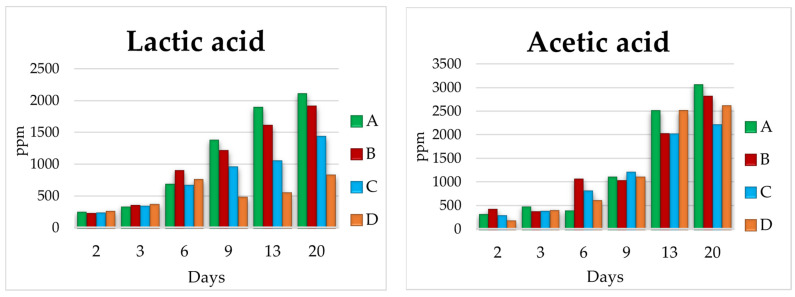
Lactic acid and acetic acid evolution during the fermentation of the four experimental beers in 1 L (A, B, C, D). A, sequential experiment; B, (mixed *L. brevis* + S-04) + B6; C, *L. brevis* + (mixed S-04 + B6); D, mixed experiment *L. brevis* + S-04 + B6.

During the fermentation performed by the different experiments (A, B, C, D), the evolution of lactic and acetic acid in them was also evaluated (Figure 4).

The first three days of the different fermentations show no significant changes were observed between them. However, it was from day six onwards that the greatest differences began to be observed. In experiments A and B, a progressive increase in the concentration of lactic acid and acetic acid was observed, despite the difference in bacteria inoculation day. This indicates the absence of inhibition of the bacteria by the *Saccharomyces* strain (S-04). However, the production of acetic acid in experiment A and B starts to become noticeable once the *Brettanomyces* yeast was inoculated (day 8 for beer A and day 4 for beer B). On the other hand, in experiment C, lactic acid only reached a concentration of 1424 ppm, while in experiment D it was notably lower, being only 818 ppm. This fact may be due to the competition between the different species during fermentation, both the interactions between yeasts, as well as the interactions between yeast and LAB [46,47,48].

### 3.3. Sensory Analysis

The fermentations performed for the four experiments in 1 L (A, B, C and D) were sensory evaluated after one month of maturation to determine the most suitable conditions for brewing lambic beer with the tested strains.

**Figure 5:**
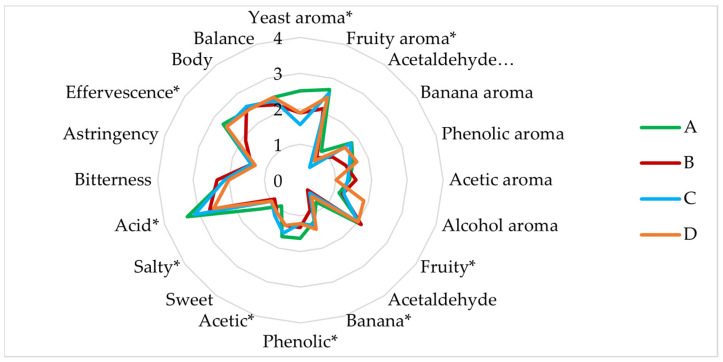
Sensory analysis of the four experimental beers A, B, C, and D fermented in 1 L. A, sequential experiment; B, (mixed *L. brevis* + S-04) + B6; C, *L. brevis* + (mixed S-04 + B6); D, mixed *L. brevis* + S-04 + B6. *, Significantly different (ANOVA; *p* < 0.05).

Figure 5 shows the individual characteristics of each of the sensory analysed beers. All beers showed average values for fruity aromas. The production of aroma-active compounds by *Brettanomyces* yeast is very complex, the most abundant being volatile phenols. Volatile phenols have very low thresholds, with a wide variety of aroma descriptors such as horse sweat, leathery, spicy, medicinal and smoky [49,50]. Therefore, these beers with phenolic notes and acetic aroma were described as farmhouse aroma beers by the panellists.

Regarding their flavour, these beers showed a medium fruity character, as well as an accentuated acidity due to the lactic and acetic acid concentrations characteristic of lambic style beers [51,52]. However, they were also characterised by sweet and salty notes, with a medium bitterness, body and balance.

All the tasted beers obtained higher scores in overall impression, although the sensory profile was different in terms of intensities.

### 3.4. Larger Scale: 14 L

The analyses performed in 1 L for experiments A, B, C and D in terms of fermentation kinetics, evolution of lactic and acetic acid concentration, as well as sensory analysis, showed that experiments A and B showed faster fermentation kinetics than experiments C and D, as well as a higher production of organic acids, characteristic of lambic beers. Likewise, despite having common characteristics with the rest of the experiments, beers A and B showed a higher fruity profile at sensory level, as well as phenolic and banana, with accentuated acidity. Based on these results, experiments A and B were finally selected for larger-scale fermentation in 14 L.

The 14 L fermentations were performed under the same fermentative conditions (aerobiosis, 20–22 °C and no stirring) and in steel milk-type fermenters. Pure culture fermentations were also performed with the *L. brevis* strain, strain S-04, strain B6 and another one with *L. brevis* and strain S-04 inoculated together. In this way it was also possible to check the development of the different microorganisms separately.

**Figure 6:**
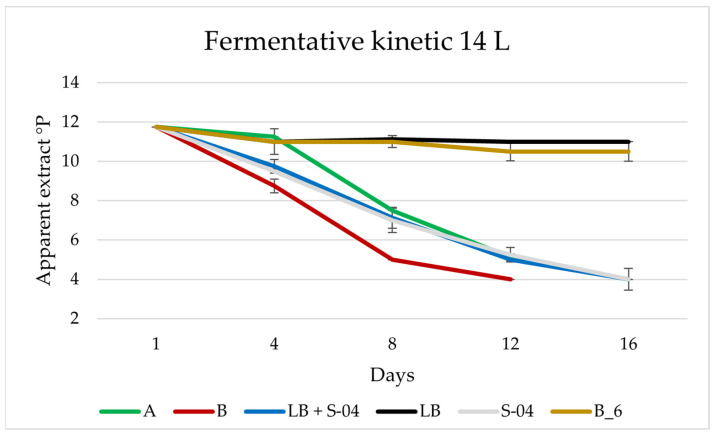
Fermentation kinetics of the experimental beers A and B fermented in 14 L, with LB + S-04 (*L. brevis* and S-04 inoculated at the same time), LB (*L. brevis*), S-04 and B6 (*B. bruxellensis*) as controls.

Figure 6 shows the fermentation kinetics of experiments A and B as well as the controls. The final apparent extract for beers A and B was 4 °P, with a fermentation duration of 12–16 days, therefore, compared to 1 L the stabilisation was prolonged by 3–4 days. This may be due to the incomplete homogenisation of nutrients under static fermentation conditions. Fermentations by the controls were similar for *L. brevis* and *B. bruxellensis* B6 strain, being very slow during this period, while the controls with *L. brevis* + S-04 and S-04 were almost similar, even though one of them contained bacteria with a final apparent extract of 4 °P.

#### 3.4.1. Cell Monitoring during 14 L Fermentation

**Figure 7:**
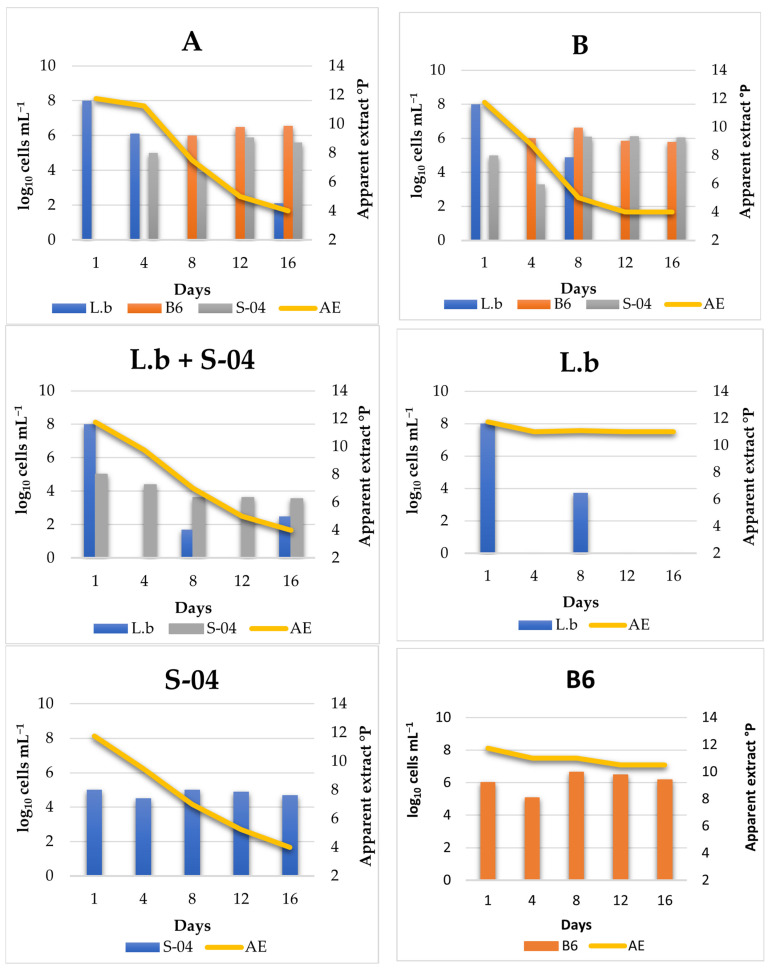
Fermentative kinetics and population monitoring of the experimental beers A and B, and controls (L.b + S-04, L.b, S-04 and B6) fermented in 14 L. The data shown are the average of three independent samples. L.b, *Lactobacillus brevis*; B6, *Brettanomyces bruxellensis*; AE, apparent extract.

To determine the population evolution of the different microorganisms during fermentation, experimental fermentations A and B, as well as the controls (*L. brevis* + S-04 in mixed fermentation, *L. brevis*, S-04 and B6), were monitored (Figure 7). Monitoring was performed by plating the samples on specific culture media on different days of the fermentation process.

On day 1, *L. brevis* was added to experiment A with an inoculum size of approximately 10^8^ cells mL^−1^. It was observed that the population decreased until day 4, when strain S-04 was added (10^5^ cells mL^−1^). From this moment the concentration of bacteria decreased notably, reaching practically zero. This may be due to the fact that as the fermentation volume increases, the homogenisation is not the same and therefore there is greater competition for nutrients. It could also have been affected by the temperature if it were not reached homogeneously. This behaviour was also observed in the lactic acid bacteria control where the bacterial population decreased drastically. On day 8, the *Brettanomyces* B6 strain (10^6^ cells mL^−1^) was inoculated and its population remained stable throughout the fermentation. However, once the *Brettanomyces* yeast was added, the *Saccharomyces* S-04 strain increased its population again (from 8 × 10^3^ to 7.7 × 10^5^ cells mL^−1^). This may be due to the slower metabolism of *Brettanomyces* yeasts [7], as well as inter-species competition, as observed in other studies of sequential fermentations with non-*Saccharomyces* strains [53].

#### 3.4.2. Beer Analysis

**Figure 8:**
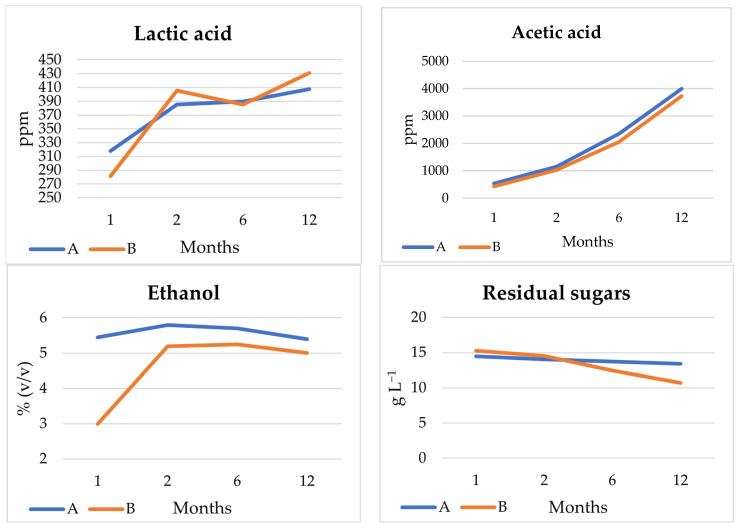

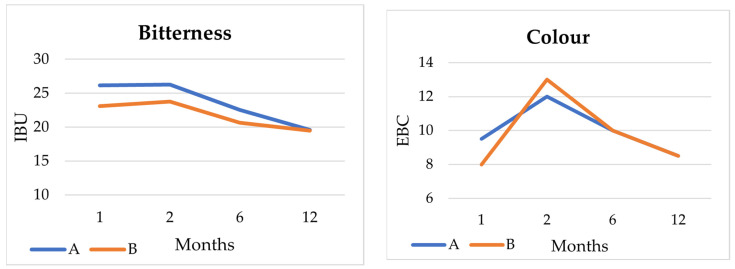
Evolution of the main parameters analysed in beers A and B at 1, 2, 6 and 12 months of aging.

Once fermentation was finished, the experimental beers A and B were bottled and matured at 14 °C. To determine their evolution, beers were analysed on different maturation times of 1, 2, 6 and 12 months (Figure 8).

The production of lactic acid in 14 L, as well as its evolution over the months, was much lower than that obtained in 1 L, mainly due to the population level of bacteria, which remained very low or null during fermentation. Levels were therefore lower than those that could be found in commercial beers [10]. On the other hand, the production of acetic acid by *Brettanomyces* yeast was remarkable, going from 535 and 425 ppm for experiments A and B, to 3994.5 and 3728.5 ppm, respectively, after 12 months. This increase in acetic acid concentration has also been observed in other studies of spontaneous fermented beers analysed at different maturation times [54]. Acetic acid is a flavour that is not normally sought after in beer as it is not desired by many consumers, however it is considered a positive characteristic in spontaneous fermented barrel aged beers such as lambic, gueuze, and Flanders [55]. Thus, one of the main characteristics of lambic beers is the high concentrations of this organic acid [47]. Its production depends on the strain and the initial oxygenation of the wort and can vary in some studies between 400 and 1200 ppm [47], while in others from 5000 to 20,000 ppm [56].

*Brettanomyces* yeasts are able to ferment the complex sugars left residually during fermentation [47]. This behaviour was observed during the first two months of maturation with an increase in ethanol content in both beers, being more noticeable in beer B, as due to possible competition between *Saccharomyces* and *Brettanomyces*. At the time of bottling the ethanol content was low (less than 3% (*v*/*v*). Subsequently, the ethanol was slightly reduced, with final concentrations of 5.4% (*v*/*v*) (experiment A) and 5% (*v*/*v*) (experiment B). Similarly, variations in sugar concentrations were related to ethanol production, with the greatest decrease in beer in experiment B (from 15.25 g L^−1^ in month 1 to 10.7 g L^−1^ in month 12). This fact has been observed in commercial lambic beers due to the high attenuation of *Brettanomyces* yeasts [10].

During the ageing process of the beers the iso-α-acids present in the beer are degraded to 4-methylpentan-2-one and 3-penten-2-one [57], which is the reason beers A and B showed a decrease in concentration from 26.15 to 19.6 IBU in beer A, and from 23.1 to 19.5 IBU in beer B, resulting in a more palatable beer. A negative correlation between bitterness and acetic acid (r = 0.84, *p* < 0.01) was also observed.

Some studies suggest that the increase in colour of beers during ageing is due to Maillard reactions and oxidation of polyphenols and may also be related to an increase in the concentration of the compound furfural [57]. The furfural was only detected in beer A at two months of maturation, thus this increase in colour may or may not be related. On the other hand, the increase or decrease in colour can be caused by reversible or non-reversible oxidation/reduction reactions of the pigments. The formation of oxidative radicals requires an oxidative reducing compound that also acts as a radical scavenger [58]. Therefore, the decrease in colour due to the oxidation of compounds could be related to the increase in the antioxidant capacity of the beers over time.

Finally, it should be noted that the final pH of the beers after 12 months of ageing, as a consequence of the increase in acetic acid, was around 2.5–3 for both beers, being slightly lower than commercial beers [4,10].

#### 3.4.3. Volatile Compounds

The aroma compounds produced by beers A and B were analysed at 1, 2, 6 and 12 months of maturation to see how they evolved over time (Table 2).

From a general point of view, the total higher alcohols have been increasing over the months, from 45.04 to 79.31 mg L^−1^ for beer B and from 52.36 to 65.19 mg L^−1^ for beer B. The same evolution was observed in studies performed by Bossaert et al. [54], where they analysed beers during nine months of maturation. However, the concentrations obtained are lower than those observed in previous studies with *Saccharomyces* and non-*Saccharomyces* yeasts [59,60]. Isobutanol, methionol and β-phenylethanol were the main higher alcohols detected, the evolution of β-phenylethanol being remarkable, as it increased fivefold from 2.81 to 10.57 mg L^−1^ for beer A and from 2.86 to 9.45 mg L^−1^ for beer B. The production of methionol is also noteworthy, since despite not exceeding the threshold (2 mg L^−1^) [61], 12-month beers have increased significantly and were close to this threshold, with a concentration of 1.36 mg L^−1^ for beer A and 1.30 mg L^−1^ for beer B. Methionol is a sulphur volatile compound formed during fermentation by yeasts or bacteria [62]. It provides sweet, raw potato and oiled cabbage flavours and it is generally found at high concentrations in top-fermented wheat beers [63]. The compound 1-hexanol has been previously reported in beer, but not lambic beer [64].

The second most important group of aromatic compounds are esters, which contribute to the fruity flavour in beer. An increase in concentration of esters was also observed, however, none above their threshold. This was also observed in studies on the ageing of spontaneous fermented beer, especially in ethyl hexanoate and ethyl octanoate compounds [54]. The volatile compounds detected were mainly ethyl isovalerate, isoamyl acetate, ethyl hexanoate, ethyl octanoate and 2-phenylethyl acetate. The production of ethyl octanoate stood out, as its concentration increased by a factor of 10 (from 0.02 to 0.24 mg L^−1^ in beer A, and from 0.01 to 0.26 mg L^−1^ in beer B) and the concentration of isoamyl acetate was only detected in 12-month-old beers (0.07 mg L^−1^ in beer A and 0.08 mg L^−1^ in beer B). Ethyl octanoate has been previously reported in the literature as being found in lambic beer with a concentration of 0.16–0.59 mg/L [9], being similar to the concentration obtained (0.24–0.26 mg L^−1^).

*Brettanomyces* yeast is able to produce high concentrations of ethyl esters (such as ethyl acetate, hexanoate and octanoate) which contribute to tropical fruit and pineapple-like flavour, but lower concentrations of other acetate esters such as isoamyl acetate, due to its esterase activity [65,66]. Nevertheless, the concentration of isoamyl acetate detected at 12 months was found to be well below the threshold. Likewise, the final fermentation and maturation stages of lambic-style beers are characterised by low concentrations of esters [67]. Lambic beers are also characterised by ethyl lactate concentrations above 400 mg L^−1^ [68]. Ethyl lactate is formed from lactic acid, and in this trial the amounts of lactic acid were lower than those obtained in 1 L, as a consequence of the low activity of lactic acid bacteria. Therefore, no ethyl lactate concentration was detected in the samples.

Regarding the evolution of fatty acids, a progressive increase was observed over the months, being particularly remarkable in the production of isovaleric acid, which was not detected until month 12 (5.59 mg L^−1^ for beer A and 4.72 mg L^−1^ for beer B), also exceeding the threshold (2.5 mg L^−1^) [69]. The *Brettanomyces* genus has been found to produce a large amount of fatty acids that contribute to unpleasant rancid aromas and flavours, with isovaleric acid being the most produced [70]. However, these acids are esterified over time, thus these aromas become less noticeable [66]. *Brettanomyces* is able to esterify medium and long chain fatty acids (C9 C10, C14 and C16) that contribute cheese and rancid flavours, into corresponding esters, producing sweet, grape, apple and wine-like flavour changes [51]. Thus, the increase in octanoic acid also led to an increase in ethyl octanoate over the months.

Diacetyl is a compound that can be found in lambic beers, however, in the study performed by Witrick et al. on different samples taken throughout the maturation of a gueuze beer (3, 6, 9, 12, 28 months), it was not detected in any of the beers [33]. This is why in this study diacetyl values above the threshold were only detected in the 2-month-old beers, which then decreased over the following months and finally became non-detectable (0.33 mg L^−1^ for beer A, and 0.20 mg L^−1^ for beer B).

Finally, regarding the production of volatile phenols, lambic beers are associated with flavour and aroma descriptors such as horse sweat, leathery, spicy, medicinal and smoky, with very low detection threshold [49,50]. The production of volatile phenols by *Brettanomyces* is a two-step conversion, a decarboxylation and a reduction step, acting on the ferulic and p-coumaric acids present in the wort [71]. Production will therefore depend mainly on the strain selected. The compound analysed in this study was guaiacol, whose concentration increased over the 12-month period from 0.03 to 0.09 mg L^−1^ for beer A and from 0.03 to 0.06 mg L^−1^ for beer B, being in both cases above the threshold (3.8 µg L^−1^) [72]. Guaiacol was correlated with total higher alcohols production positively r = 0.95, *p* < 0.01.

In general, the volatile compounds found in these beers can be found in commercial lambic beers [4,10].

#### 3.4.4. Melatonin Production

All species of microorganisms (such as bacteria, yeasts and fungi) may have the ability to produce melatonin, as it is a biosynthetic mechanism conserved throughout the evolution of species. It also has the ability to preserve products (food or beverages) over time, as well as regulating the circadian people rhythms. Melatonin therefore acts as a strong antioxidant which is degraded in the presence of oxygen or by free radicals in the environment during the storage of fermented foods [73].

**Figure 9:**
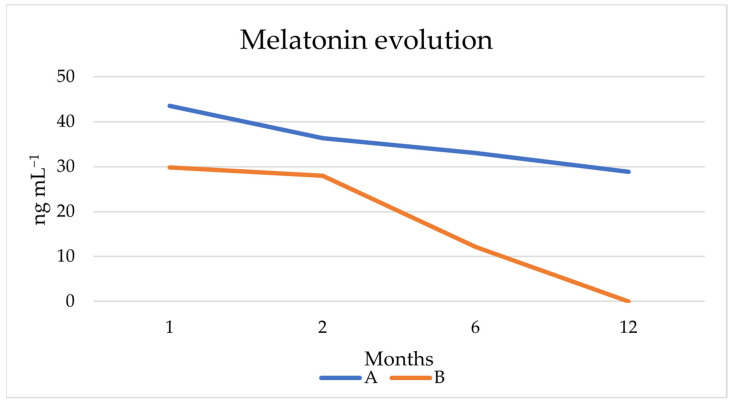
Melatonin production by the two experimental beers A and B in 14 L fermentation during aging.

Figure 9 shows the evolution of melatonin over the 12 months of the study. As can be seen, its concentration decreased over time, finally becoming undetectable for beer B, while for beer A it went from 43.56 to 28.87 ng mL^−1^. These values are higher compared to the melatonin content that can be found in other beers or foods [37], and similar to others studied in non-conventional yeasts [53,59,60,74].

#### 3.4.5. Antioxidant Capacity

Antioxidant capacity provides considerable information on oxidation resistance, quantitative contribution of antioxidant molecules and total antioxidant capacity of the food (Table 3).

Antioxidant capacity is related to parameters such as total phenolic and flavonoid content, DPPH radical scavenging activity and ABTS radical cation scavenging activity [75]. Yeasts belonging to the genera *Brettanomyces* are characterised by a strong production of phenolic compounds through specific enzymatic activities [76]. This is why beers with a high phenolic content contribute to the increase in the antioxidant capacity of the beer, making the beer a highly dietary consumable beverage. In this study, the production of guaiacol was studied as phenolic compounds, so further studies would be necessary to be able to contrast the slight increase observed in the antioxidant capacity over the months (from 9.91 to 13.03 mmol TE L^−1^ for beer A, and from 9.93 to 15.56 mmol TE L^−1^ for beer B). However, it should be noted a positive correlation was observed between acetic acid production and increased antioxidant capacity (r = 0.91, *p* < 0.01). It also showed negative correlations with bitterness (r = 0.81, *p* < 0.05) and melatonin production (r = 0.75, *p* < 0.05).

#### 3.4.6. Sensory Analysis

**Figure 10:**
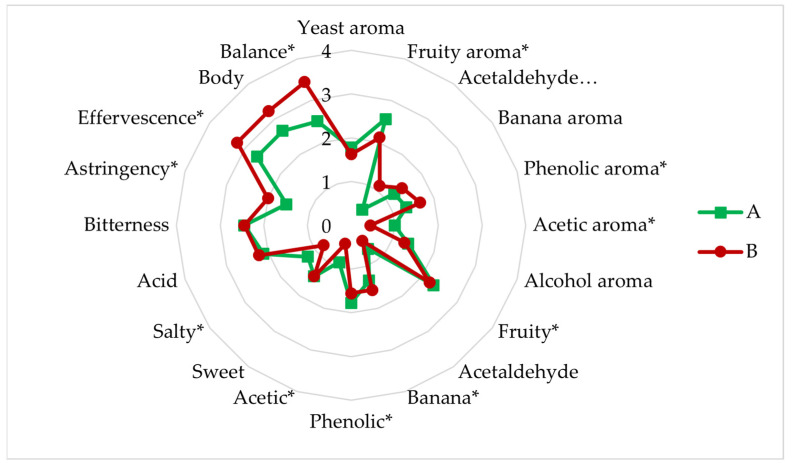
Sensory analysis of the experimental beers A and B fermented in 14 L. *, Significantly different (ANOVA; *p* < 0.05).

Beers A and B fermented in 14 L were sensory evaluated after 12 months of maturation to determine their evolution. Figure 10 shows the main parameters analysed for aroma and flavour.

Compared to beers A and B fermented in 1 L after one month of maturation, the level of acidity detected is noteworthy, mainly due to the low production of lactic acid by the lactic bacteria. As seen in the analysis of aroma compounds, the esters have not changed much, except for beer A from six months to 12 months, however, both beers were rated as having a medium fruity aroma in the tasting, as well as the beers fermented in 1 L. This may be due to the “matrix effect” that aromas can experience in beer, as synergistic or antagonistic interactions can occur between aromas. Thus, the perception and taste of beer can be modified despite the fact that the aromas are below the threshold [49,77,78,79,80,81]. Beer B stood out from beer A in terms of acetaldehyde, banana and phenolic aroma and less salty and acetic flavour. On the other hand, it stood out notably in terms of astringency, effervescence and balance, which is why it was more highly rated than beer A overall. As in the 1 L trials, the tasting panel noted the so-called “Brett flavour” (barnyard, clove, horsy, leathery, medical, mousy, smoky, and spicy) [47] in both beers. Beers fermented in 14 L and matured for 12 months were rated better overall than beers fermented in 1 L and matured for 1 month, thus that maturation over time flavours the palatability of these beers.

#### 3.4.7. Statistical Analysis

**Figure 11:**
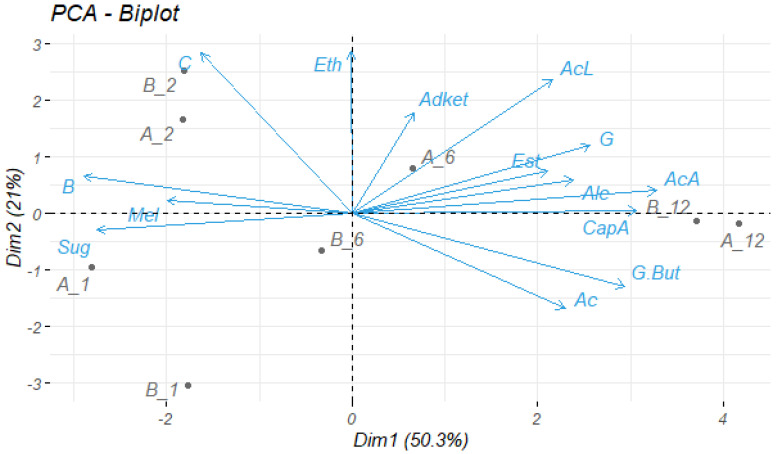
Projection of the experimental beers A and B (14 L) on the axes formed by the principal components 1 and 2. Each object is the average of the three corresponding experimental beers. Eth, ethanol; AcL, lactic acid; AcA, acetic acid; C, colour; B, bitterness; Sug, residual sugars; Alc, total higher alcohols; Est, total esters; Ac, total fatty acids; AdKet, total aldehydes/ketones; G. But, γ-butyrolactone; G, guaiacol; Mel, melatonin; CapA, antioxidant capacity. A_1 and B_1, beer A and B after one month; A_2 and B_2, beer A and B after two months; A_6 and B_6, beer A and B after six months; A_12 and B_12, beer A and B after 12 months.

Principal component analysis (PCA) was applied to assess trends in the data and determine whether experimental beers A and B group according to their maturation with respect to the main parameters analysed (volatile compounds, lactic acid, acetic acid, colour, bitterness, ethanol, residual sugars, antioxidant capacity and melatonin production). Two principal components (PCs) were extracted from the seven dimensions, representing 71.3% of the values. PC1 (Dim1) explained up to 50.3% of the total variance and PC2 (Dim2) explained another 21% (Figure 11).

Since the differences between beers A and B over the months were not greatly different, the beers were grouped in different quadrants, but always according to the month of maturation, except for those of month 6, which were placed in opposite quadrants. The one-month-old beers were in the negative quadrants of both components, characterised by their sugars content. The two-month-old beers were on the positive side of PC2, characterised mainly by the increase in colour that occurred in this month. Following the order of maturation, the successive beers would be those corresponding to 6 months, where beer A was located in the positive zone of both components, characterised by the production of aldehydes/ketones, lactic acid, acetic acid, guaiacol, esters and total higher alcohols, while beer B was conversely located in the negative zone of PC1 and PC2, not being defined by any of the analysed compounds. Finally, the beers analysed after 12 months of maturation were positioned in the positive zone of PC1, defined mainly by their antioxidant capacity, the production of γ-butyrolactone and total fatty acids.

## 4. Conclusions

This study aimed to conduct a first approach to the controlled fermentation of lambic beer. Different microorganisms interact in lambic beers, thus different combinations of microorganisms were used to determine which was the most suitable for brewing lambic beer. *Brettanomyces* yeasts have been identified in spontaneous beer fermentations, being one the most important factors that influence the lambic beer style.

Beers obtained from combinations A (sequential) and B ((*L. brevis* + *Saccharomyces*) + *B. bruxellensis*) showed faster fermentation kinetics, produced higher lactic and acetic acid concentrations and were sensorially more acceptable than the other experimental combinations. After 12 months of maturation, both beers showed an aromatic profile with fruity and “Brett” flavour (barnyard, clove, horsy, leathery, medical, mousy, smoky and spicy), where the concentration of higher alcohols and esters increased over the months. An increase in lactic and acetic acid and a decrease in bitterness were also observed. Acetic acid is a compound that is not generally accepted by consumers but is considered to be a widely accepted characteristic of this style of beer (Lambic, gueuze and Flanders). The final ethanol concentration was 5.4% (*v*/*v*) for beer A and 5% (*v*/*v*) for beer B.

Beers fermented and tasted after 12 months of maturation were rated better than those brewed in 1 L scale and tasted after one month of maturation, thus maturation favours the palatability of these beers. The antioxidant capacity of these beers also increased over the months of maturation.

## Figures and Tables

**Table 1:** Microorganism strains used for the study.

Microorganism	Strain	Origin
*Brettanomyces bruxellensis*		Doemens, Munich, Germany
*Brettanomyces lambicus*		Doemens, Munich, Germany
*Brettanomyces bruxellensis*	RS15/CR002_1 (B1)	D.O. “Ribeira Sacra”, Galicia, Spain
*Brettanomyces bruxellensis*	RS16/CR001-T2_1 (B2)	D.O. “Ribeira Sacra”, Galicia, Spain
*Brettanomyces bruxellensis*	RS16/CR003_1 (B3)	D.O. “Ribeira Sacra”, Galicia, Spain
*Brettanomyces bruxellensis*	RS16/CR003-T2 (B4)	D.O. “Ribeira Sacra”, Galicia, Spain
*Brettanomyces bruxellensis*	RS17/CR020 (B5)	D.O. “Ribeira Sacra”, Galicia, Spain
*Brettanomyces bruxellensis*	RS17/CR028 (B6)	D.O. “Ribeira Sacra”, Galicia, Spain
*Saccharomyces cerevisiae*	S-04	Fermentis, Lesaffre, Marcq-en-Barœul France
*Lactobacillus brevis*		Doemens, Munich, Germany

**Table 2:** Volatile compounds found in Lambic beers in 14 L (mg L**^−^**^1^).

Maturation Times	1 Month		2 Months		6 Months		12 Months	
	A	B	A	B	A	B	A	B
Isobutanol	7.03 ± 0.39 ^b^	7.86 ± 1.10 ^b^	8.04 ± 0.30 ^b^	8.13 ± 0.02 ^ab^	7.68 ± 0.23 ^b^	7.59 ± 0.08 ^b^	11.27 ± 1.61 ^a^	10.04 ± 0.97 ^ab^
Isoamyl alcohol	35.12 ± 2.17 ^b^	41.56 ± 9.84 ^ab^	45.26 ± 1.10 ^ab^	44.19 ± 0.45 ^ab^	45.81 ± 0.37 ^ab^	44.97 ± 2.34 ^ab^	55.95 ± 2.88 ^a^	44.28 ± 6.85 ^ab^
1-hexanol	nd	0.01 ± 0.00 ^b^	nd	nd	nd	nd	0.14 ± 0.02 ^a^	0.12 ± 0.02 ^a^
Methionol	0.08 ± 0.01 ^b^	0.07 ± 0.00 ^b^	0.10 ± 0.01 ^b^	0.10 ± 0.01 ^b^	0.05 ± 0.08 ^b^	nd	1.36 ± 0.01 ^a^	1.30 ± 0.19 ^a^
β-phenylethanol	2.81 ± 0.26 ^b^	2.86 ± 0.04 ^b^	4.48 ± 0.09 ^b^	4.76 ± 0.65 ^b^	4.50 ± 0.40 ^b^	3.93 ± 0.33 ^b^	10.57 ± 1.36 ^a^	9.45 ± 0.82 ^a^
**Total higher** **alcohols**	45.04 ± 2.84	52.36 ± 10.98	57.88 ± 1.50	57.18 ± 1.13	47.24 ± 1.28	42.50 ± 2.76	79.29 ± 5.88	65.19 ± 8.85
Ethyl butyrate	nd	nd	nd	nd	nd	nd	0.13 ± 0.01 ^a^	0.06 ± 0.09 ^a^
Ethyl isovalerate	0.08 ± 0.01 ^a^	0.09 ± 0.00 ^a^	0.08 ± 0.09 ^a^	0.08 ± 0.00 ^a^	0.09 ± 0.00 ^a^	0.09 ± 0.00 ^a^	0.08 ± 0.02 ^a^	0.07 ± 0.00 ^a^
Isoamyl acetate	nd	nd	nd	nd	nd	nd	0.07 ± 0.01 ^a^	0.08 ± 0.01 ^a^
Ethyl hexanoate	0.04 ± 0.00	0.41 ± 0.53	0.05 ± 0.00	0.04 ± 0.00	0.06 ± 0.00	0.02 ± 0.03	0.10 ± 0.12	0.04 ± 0.01
Ethyl octanoate	nd	nd	0.02 ± 0.00	0.01 ± 0.00	0.02 ± 0.00	0.02 ± 0.00	0.24 ± 0.01	0.26 ± 0.03
2-phenylethyl acetate	nd	0.00 ± 0.00 ^b^	0.00 ± 0.00 ^b^	0.00 ± 0.00 ^b^	0.00 ± 0.00 ^b^	0.00 ± 0.00 ^b^	0.01 ± 0.00 ^a^	0.01 ± 0.00 ^a^
**Total esters**	0.12 ± 0.01	0.50 ± 0.53	0.15 ± 0.09	0.13 ± 0.00	0.17 ± 0.00	0.13 ± 0.03	0.63 ± 0.20	0.52 ± 0.14
Butyric acid	0.02 ± 0.03 ^b^	nd	0.15 ± 0.01 ^b^	0.05 ± 0.08 ^b^	0.07 ± 0.00 ^b^	0.06 ± 0.00 ^b^	2.04 ± 0.05 ^a^	1.94 ± 0.44 ^a^
Isovaleric acid	0.00 ± 0.00 ^b^	0.00 ± 0.00 ^b^	0.01 ± 0.00 ^b^	0.01 ± 0.00 ^b^	0.01 ± 0.00 ^b^	0.01 ± 0.00 ^b^	**5.59 ± 0.20 ^a^**	**4.72 ± 1.28 ^a^**
Hexanoic acid	0.67 ± 0.07 ^b^	0.93 ± 0.03 ^b^	0.79 ± 0.18 ^b^	0.73 ± 0.00 ^b^	0.93 ± 0.23 ^b^	1.09 ± 0.06 ^ab^	1.58 ± 0.07 ^a^	1.60 ± 0.21 ^a^
Octanoic acid	0.97 ± 0.09	1.99 ± 0.07	1.28 ± 0.37	1.22 ± 0.03	1.60 ± 0.75	2.12 ± 0.06	1.81 ± 0.50	2.30 ± 0.12
Decanoic acid	0.10 ± 0.04 ^ab^	0.07 ± 0.00 ^b^	0.18 ± 0.03 ^ab^	0.14 ± 0.03 ^ab^	0.16 ± 0.01 ^ab^	0.14 ± 0.03 ^ab^	0.23 ± 0.07 ^a^	0.22 ± 0.06 ^ab^
**Total fatty** **acids**	1.77 ± 0.17	3.00 ± 0.11	2.41 ± 0.60	2.15 ± 0.08	2.77 ± 0.97	3.41 ± 0.09	11.26 ± 0.49	10.78 ± 2.11
Diacetyl	nd	nd	**0.33 ± 0.03**	**0.20 ± 0.29**	**0.18 ± 0.02**	nd	nd	nd
Furfural	nd	nd	0.02 ± 0.01 ^a^	nd	nd	nd	nd	nd
Acetoin	nd	nd	nd	nd	nd	nd	nd	0.45 ± 0.01 ^a^
Benzaldehyde	0.01 ± 0.01	0.04 ± 0.01	0.02 ± 0.02	0.01 ± 0.01	0.04 ± 0.02	0.04 ± 0.01	nd	nd
**T. aldehydes/** **ketones**	0.01 ± 0.01	0.04 ± 0.01	0.35 ± 0.05	0.22 ± 0.30	0.22 ± 0.04	0.04 ± 0.01	nd	0.45 ± 0.01
**γ-Butyrolactone**	2.62 ± 0.00 ^b^	2.62 ± 0.00 ^b^	nd	nd	2.62 ± 0.00 ^b^	2.62 ± 0.00 ^b^	7.20 ± 0.24 ^a^	6.67 ± 0.34 ^a^
**Guaiacol**	**0.03 ± 0.00**	**0.03 ± 0.00**	**0.05 ± 0.01**	**0.05 ± 0.00**	**0.05 ± 0.01**	**0.03 ± 0.04**	**0.09 ± 0.05**	**0.06 ± 0.00**

Data are means ± standard deviations of three independent samples. Compounds above their threshold levels are marked in bold. Data with different superscript letters within each row are significantly different (Tukey tests: *p* < 0.05). nd = not detected.

**Table 3:** Evolution of antioxidant activity of the experimental beers A and B in 14 L fermentation during 12 months.

Yeast Strains		Q1	Q2	Qt
**1 month**	**A**	3.41 ± 0.68 ^a^	6.50 ± 2.40 ^a^	9.91 ± 3.09 ^a^
	**B**	3.61 ± 0.37 ^a^	6.32 ± 0.39 ^a^	9.93 ± 0.76 ^a^
**2 months**	**A**	2.78 ± 0.16 ^a^	6.28 ± 0.48 ^a^	10.06 ± 0.32 ^a^
	**B**	3.62 ± 0.21 ^a^	6.57 ± 0.96 ^a^	10.19 ± 1.17 ^a^
**6 months**	**A**	4.09 ± 0.47 ^a^	8.12 ± 0.84 ^a^	12.20 ± 1.31 ^a^
	**B**	4.02 ± 0.93 ^a^	7.46 ± 1.80 ^a^	11.48 ± 2.73 ^a^
**12 months**	**A**	4.31 ± 0.06 ^a^	8.72 ± 0.34 ^a^	13.03 ± 0.40 ^a^
	**B**	4.31 ± 0.39 ^a^	11.25 ± 4.20 ^a^	15.56 ± 3.81 ^a^

Data are means ± standard deviations of three independent samples expressed as millimoles of Trolox equivalents per litre (mmol TE L^−1^). Q1, fast-acting antioxidants, Q2, slow-acting antioxidants, Qt, total antioxidants. Data with different superscript letters within each column are significantly different (Tukey tests: *p* < 0.05).

## Data Availability

Not applicable.
